# Elucidation of *in vitro* cellular steps induced by antitumor treatment with plasma-activated medium

**DOI:** 10.1038/s41598-019-41408-6

**Published:** 2019-03-19

**Authors:** Julie Chauvin, Laure Gibot, Elena Griseti, Muriel Golzio, Marie-Pierre Rols, Nofel Merbahi, Patricia Vicendo

**Affiliations:** 1 Université de Toulouse, UPS, CNRS, Laplace UMR CNRS, 5213 Toulouse, France; 2 Institut de Pharmacologie et Biologie Structurale, IPBS, Université de Toulouse, CNRS, UPS, Toulouse, France; 3 Université de Toulouse; UPS, CNRS, IMRCP, 118 route de Narbonne, F-31062 Toulouse, Cedex 9 France

## Abstract

Numerous studies have reported cold atmospheric plasma cytotoxic activities in various cancer cell lines, either by direct exposure to non-thermal plasma or indirectly by activating a medium (plasma-activated medium, PAM) prior to cell treatment. We suggested the use of *in vitro* 3D tumor model spheroids to determine the potential of PAM for cancer therapy at the tissue scale, especially in human tumor tissue. This work aimed to better understand the effect of PAM on human colorectal tumor spheroids by describing the *in vitro*-induced-cell death kinetics and associated mechanisms to further improve its therapeutic potential. Tumor spheroid growth was delayed depending on contact time with PAM. Medium osmolarity was increased by activation with low temperature Helium plasma jet but it did not fully explain the observed growth delay. PAM impaired tumor cell viability through intracellular ATP depletion, leading within hours to both cell apoptosis and necrosis as well as mitochondrial oxidative stress. When successive treatments were spaced over time, cumulative effects on the growth delay of spheroids were observed. Taken together, these results demonstrated that plasma-activated liquids may represent a novel and efficient therapeutic method for the treatment of tumors, especially when successive treatments are applied.

## Introduction

In 2012, 1.4 million new cases of colorectal cancer and 700 000 associated deaths were registered, making it the third most diagnosed cancer worldwide^1^. Currently, the main effective treatments are surgery associated with chemotherapy and/or radiotherapy. However, the side effects and drug resistance of these cancer therapies are responsible for a lack of efficacy. To counteract these negative effects, new methods to treat colorectal cancers involving the production of a high level of reactive oxygen species (ROS) have emerged^2,3^.

Under physiological conditions, ROS, controlled by antioxidant molecules, are essential for maintaining cellular functions and homeostasis. In contrast, under pathological processes such as colorectal cancer, there is an excessive level of ROS due to an imbalance between ROS and nonenzymatic and enzymatic antioxidants. Numerous studies have shown that a high level of ROS over the toxic threshold can lead to the preferential killing of cancer cells while not affecting normal cells^4^.

In this area of ROS anticancer therapy, cold atmospheric plasmas have received growing interest. Indeed, for biomedical applications, atmospheric pressure plasma in various configurations has been designed, such as the Dielectric Barrier Discharge (DBD)^5–7^ and corona discharge^8,9^, with different carrier gases such as argon^10,11^ or helium^12–14^, without or with oxygen^15–17^.

Their first medical applications of these plasmas were in the disinfection and healing of wounds, but plasmas are now increasingly studied for their anti-cancer properties. Several studies have reported their cytotoxic activities in various cancer cell lines, such as breast cancer^10,12^, oral cavity squamous cell carcinoma^15^, ovarian clear-cell carcinoma^11^, prostate cancer^16^, head and neck cancer^18–20^ and colorectal cancer^13,21^.

Two main approaches are being investigated in plasma cancer treatment, namely, directly exposing cells or tissue to the plasma jet^22,23^ or indirectly by activating a medium that will later be put into contact with tumors cells^4,10,13,20,24^. Plasma-activated medium (PAM) offers the advantage of being produced beforehand as a “drug” that can be stably stocked for several days^13^ and is easily usable for direct injection into target tissues. PAM can effectively kill tumor cells or tissues just as direct plasma exposure does. Plasma, as the fourth state of matter, reacts with its environment and produces reactive oxygen and nitrogen oxide species (RONS). RONS, such as hydrogen peroxide, nitrite and nitrate, have been identified as reactive species playing a major role in cancer cell death^4,10,14,25,26^. However, even if the PAM anti-cancer activities are mainly due to the presence of species such as H_2_O_2_, NO_3_^−^, or NO_2_^−^, the presence of RONS is not sufficient to fully explain the cytotoxic effects of PAM. Most of the *in vitro* studies performed on cell culture monolayers showed that PAM mainly induced cell death *via* apoptosis induction^10,11,15,17,21^. Recently, *in vivo* studies demonstrated that PAM is able to significantly reduce xenograft tumors in mouse models of pancreatic and ovarian cancers^27,28^. Due to these aspects, PAM may be considered as a potential anti-cancer agent. However, the small number of *in vivo* studies carried out with PAM does not allow the therapeutic protocol for tumor treatment to be defined. To predict and improve the performance of PAM as an *in vivo* antitumor treatment, investigations carried out *in vitro* in a 3D tumor model are necessary. 3D tissue models, such as tumor spheroids, are closer to the *in vivo* context by reproducing the architecture of a tumor^29–31^ and can help to elucidate mechanisms underlying the antitumor properties of PAM. Indeed, similar to the avascular regions of tumors, spheroids present gradients of oxygen, nutrients and metabolites, producing a gradient in cell proliferation from the center, with the presence of a necrotic core, to the proliferative surface^32^. Thus, the spheroid model is a useful tool to determine the biological effects of PAM on human tumor cell growth. While tumor spheroids have been widely used in numerous fields of biology, only a few studies have investigated the cellular-induced mechanisms associated with PAM. Thus, spheroids will help determine the potential of plasma for cancer therapy at the tissue scale, especially in human tissues.

This work aimed to better understand the sequence of processes involved in the cytotoxicity of the plasma-activated medium in spheroids and to thereby provide insight into methods to increase its potential as an anticancer treatment. Experiments were performed *in vitro* in human colorectal tumor spheroids. ATP levels were measured using luminescence analysis, and fluorescence microscopy was used to detect membrane integrity, caspases activities, and the presence of superoxide in the mitochondria. Finally, the best combination of successive treatments to efficiently reduce tumor growth was investigated.

## Results

### Medium osmolarity is increased by low-temperature helium plasma jet exposure

With the settings applied to produce helium plasma-activated medium, an increase in osmolarity was observed (Table 1). Indeed, nontreated cell culture medium has an osmolarity of 295 ± 1.5 mosmol/kg while it increased to 358 ± 23 mosmol/kg after 120 seconds of exposure to a helium plasma jet. Since osmolarity can modify cell behavior, an osmolarity control was added to the subsequent experiments, named “gas alone”. It was produced by cell culture medium exposure to helium flow alone, without plasma production, for 300 seconds. Under these conditions, medium exposed to gas flow alone presented an osmolarity of 360 ± 26 mosmol/kg, similar to the osmolarity of PAM.

**Table 1:** Characterization of media exposed to plasma and gas alone in terms of pH and osmolarity (mosmol/kg) after exposition to Helium plasma jet.

	pH	Osmolarity (mosmol/kg)	Volume after exposition (±1 µL)
Medium	7,5	295 ± 2	100
Medium exposed to gas alone for 120 s	7,5	319 ± 3	92
Medium exposed to plasma for 120 s (PAM)	7,5	358 ± 23	80
Medium exposed to gas alone for 300 s	7,5	360 ± 26	80

At least 6 independent measurements were conducted. Data are expressed as mean ± SD.

Activation of medium with plasma can cause changes in the pH of the medium^33^ however, in our experimental setup, the differences in pH were negligible before and after plasma activation due to the use of a buffered cell culture solution. However, we measured that the osmolarity was significantly increased, probably due to medium evaporation because of helium flow. Interestingly, the regulation of osmolarity is a promising research path in the field of cancer treatment^34^.

### Spheroid growth is delayed by increasing contact time with PAM

**Figure 1:**
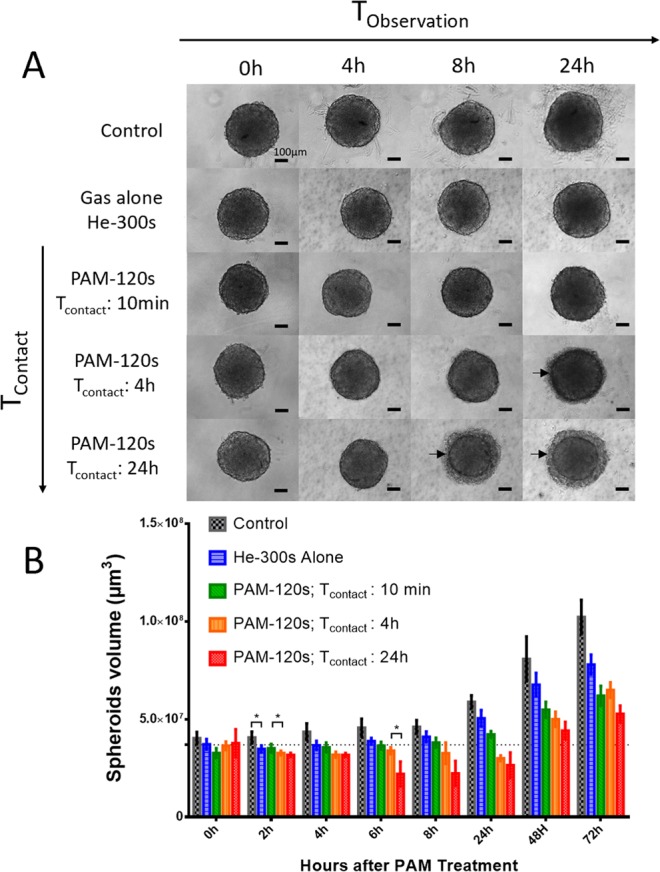
Human tumor spheroids macroscopic aspect and growth curve after PAM treatment. (**A**) Macroscopic aspect of spheroids observed by wide-field microscopy for 24 h after PAM exposition or exposition to gas alone as osmolarity control. Arrows indicate cell detachment in crown. Scale bar 100 µm. (**B**) Spheroids growth over three days after contact time T_contact_ with PAM for 10 minutes, 4 h and 24 h. *p < 0, 05.

The macroscopic aspect of spheroids was observed by wide-field microscopy over 24 h after exposure to non-activated medium (control), gas alone (osmolarity control) or PAM with different contact times, T_Contact_ (10 min, 4 h or 24 h; Fig. 1A). T_Contact_ is the duration for which spheroids were immersed in PAM. At the macroscopic scale, spheroid integrity was preserved when exposed over 24 h to medium treated with helium only (gas alone) or for 10 min with PAM, in accordance with the control condition. In contrast, when exposed to PAM for more than 10 min, the spheroid margins became blurry and started to disintegrate. Two distinct components appeared, namely, a cohesive core surrounded by a crown of detached cells. For spheroids in contact with PAM for 24 h, macroscopic effects were observed as soon as 4 h and up to 8 h posttreatment, whereas spheroids in contact with PAM for 4 h only showed deleterious effects at 24 h. Thus, the deleterious effects exerted by PAM on spheroid integrity depend on contact time.

Following the growth of the cohesive core of spheroids over three days allowed estimation of the PAM treatment effect on tumor cell growth in 3D (Fig. 1B). For conditions inducing cell detachment (i.e., T_contact_ 4 h and 24 h), evidence of spheroid decay was observed during the first 24 h, followed by a resumption of growth depending on the time of contact with PAM (Fig. 1A).

For subsequent *in vitro* experiments, the longer exposure of spheroids to PAM (T_contact_ 24 h) was chosen since this exposure time induced the most drastic effects on spheroids as early as 8 h after treatment. Interestingly, even a 5-min exposure to helium alone had no effect on the macroscopic aspects of spheroids within 24 h. After three days of culture, spheroid growth was significantly reduced compared with that of nontreated spheroids but to a less extent than that associated with PAM treatment. This result means that spheroid modifications induced by PAM are not due to a hyperosmotic effect but mainly to the generation of oxidative compounds, as reported in the literature^14,35^.

Unlike studies with cells grown in monolayers^20^, a unique PAM treatment is not sufficient to induce complete destruction of spheroids, probably due to their 3D architecture^36,37^. To optimize therapeutic protocols, an accurate description of the kinetics of the cellular mechanisms induced by PAM is needed.

### PAM impairs tumor cell viability as observed by intracellular ATP depletion and later mitochondrial dysfunction

**Figure 2:**
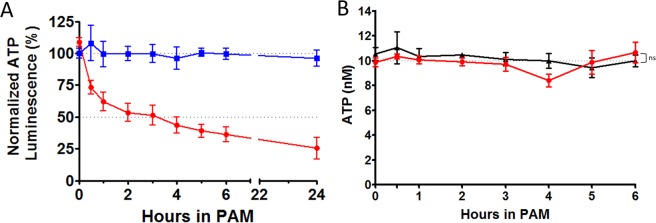
PAM treatment induced loss of ATP. (**A**) Intracellular spheroid’s content in ATP measured by luminescence over the time after exposition to PAM. Blue square: osmolarity control gas alone. Red circle: spheroids T_contact_ 24 h to PAM. (**B**) ATP stability in PAM (red circles) or in control non-activated medium (black triangle). ns: non-significant.

ATP is generally considered as a good indicator of cell viability^38,39^. For 24 h, we quantified the ATP content of spheroids after PAM treatment (T_contact_ 24 h). Figure 2A shows the spheroid’s ATP levels in PAM. The ATP was calculated based on a normalization of treated spheroids with regard to the control spheroids. While osmolarity control did not affect the ATP content of spheroids, PAM induced a significant and rapid intracellular depletion of ATP within spheroids (Fig. 2A). Two phases were observed: a fast phase during the first 3 h of exposure, leading to a 50% loss of ATP in spheroids, followed by a slower phase until reaching a 75% loss of ATP at the end of the 24 h experiment. Since PAM is known to produce reactive oxygen and nitrogen species, the stability of a standard ATP was checked when exposed to PAM in an acellular context (Fig. 2B). No effect was observed after exposure of ATP to PAM, meaning that the intracellular decrease of ATP in spheroids exposed to PAM is due to a cellular effect. To determine whether the dramatic decrease in intracellular ATP was due to ATP leakage or release, ATP was also quantified in the supernatant. Interestingly, no ATP was detected in the supernatant under these experimental conditions over the 24-h period (data not shown). This result means that either ATP is significantly consumed intracellularly or it is no longer efficiently produced by the mitochondria.

**Figure 3:**
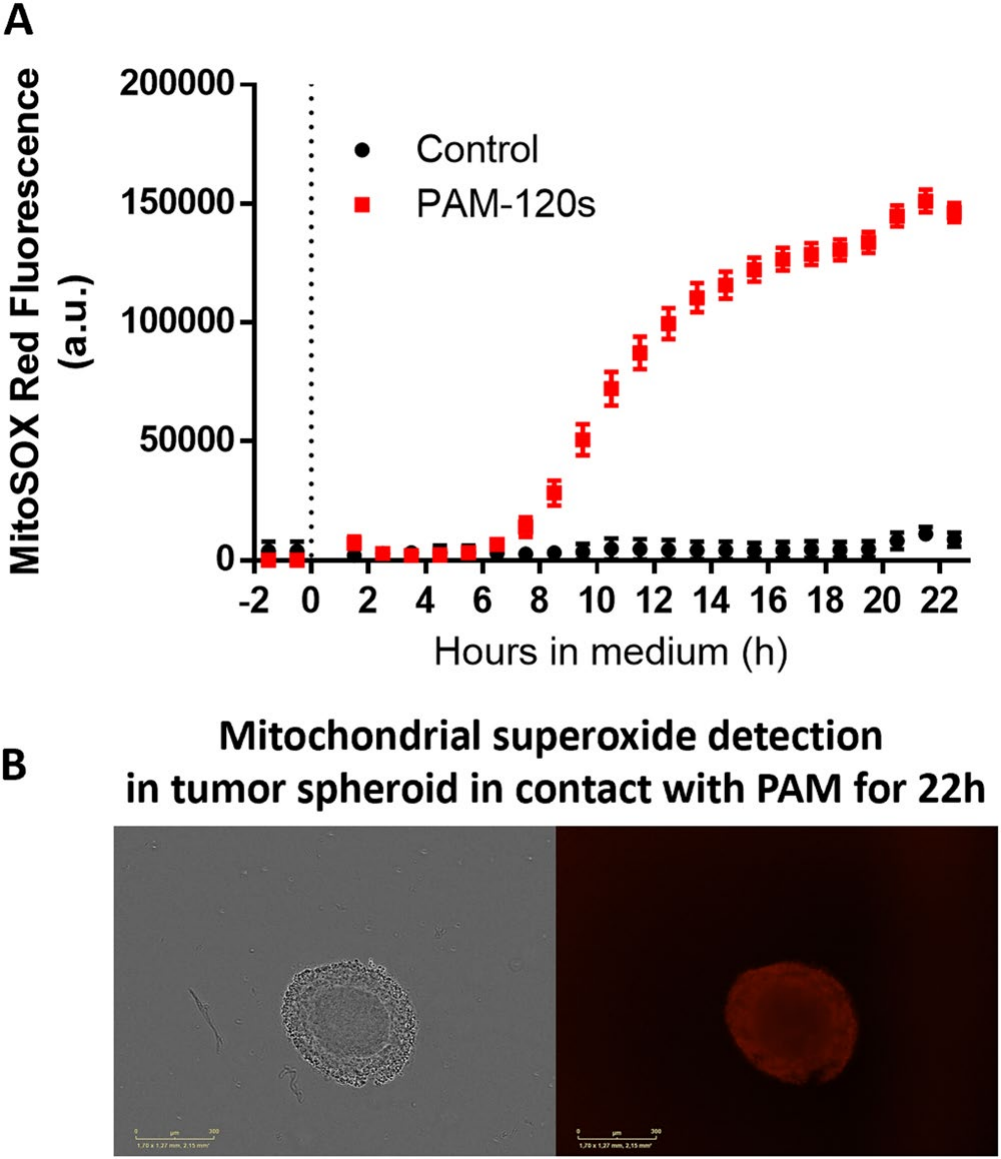
PAM treatment induced mitochondrial dysfunction through mitochondrial superoxide production. (**A**) Follow-up of the mitochondrial superoxide apparition in control and PAM-120 s spheroids over 22 h. The dotted line indicated when spheroids were exposed to PAM. (**B**) Fluorescence imaging of mitochondrial superoxide by wide field microscopy after Tcontact of 22 h to PAM. Scale bar: 300 µm.

Mitochondria dysfunction was evaluated using the MitoSOX fluorescent probe, which specifically detects the presence of mitochondrial superoxide. Interestingly, this probe is exclusively sensitive to superoxide and not to other ROS or RNS. As soon as 6 h after exposure to PAM, the MitoSOX signal significantly increased, indicating the production of mitochondrial superoxide (Fig. 3A). After 22 h of exposure to PAM, strong MitoSOX labeling was observed in the detached outer layers of the spheroid (Fig. 3B). Superoxide is known to induce oxidative stress, cellular damage and death^40^. We previously reported that the addition of superoxide dismutase (SOD) within PAM reduced DNA damage in outer spheroid layers, highlighting the key role of superoxide^13^.

### PAM induced cell death through both apoptosis and necrosis

**Figure 4:**
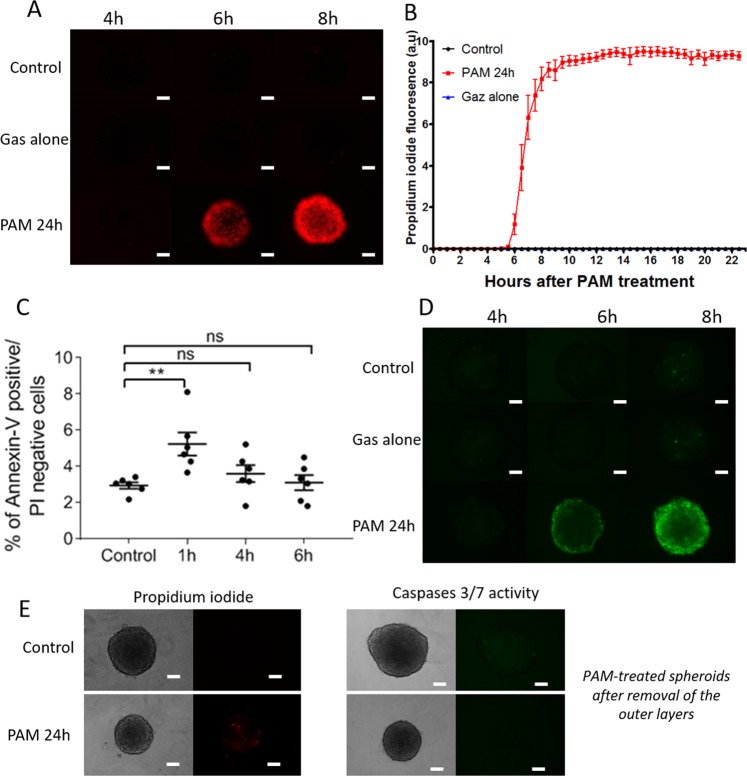
PAM treatment induced loss of plasma cell integrity and apoptosis. (**A**) Plasma membrane integrity observed thanks to propidium iodide (red) penetration. Scale bar: 100 µm. (**B**) Quantification by videomicroscopy of propidium iodide fluorescence in spheroid over the time after exposition to non-activated medium (black circles), PAM (red squares) or gas alone treatment (blue triangles). (**C**) Quantification by flow cytometry of apoptosis (Annexin-V positive/propidium iodide PI negative cells) through early externalization of phosphatidylserine in cells isolated from spheroids exposed for 1 h, 4 h or 6 h to PAM. (**D**) Caspases 3/7 activity observed by wide-field fluorescence microscopy after PAM or gas alone treatment or in control condition. (**E**) Propidium iodide penetration and caspases 3/7 activity observed by wide-field fluorescence microscopy 24 h after exposure to PAM in cohesive spheroid’s core. Crown of detached cells was removed by gentle pipetting. Scale bar: 100 µm. **p < 0.005, ns: non-significant.

Plasma membrane integrity was checked over time with a non-permeant fluorescent probe: propidium iodide. When the plasma membrane is altered, propidium iodide penetrates cells and intercalates in DNA, becoming highly fluorescent due to the increase in its fluorescence quantum yield. Non-selective permeability of plasma membranes is mainly a hallmark of dying cells. Figure 4A shows that membrane integrity was lost between 4 and 6 h of exposure to PAM. An analysis using videomicroscopy allowed real-time analysis of propidium iodide penetration (Fig. 4B and Movie 1). This analysis confirmed that membrane dysfunction is a very rapid phenomenon occurring approximately 6 h after exposure to PAM. Osmolarity control (gas alone) did not affect membrane integrity. As mentioned, a loss of plasma membrane integrity is mainly associated with cell death by both necrosis and apoptosis. Apoptosis was first assessed by quantifying the early externalization of phosphatidylserine by flow cytometry in propidium iodide negative cells (Fig. 4C). Cells in spheroids exposed to PAM displayed a rapid, i.e., within the first hour of treatment, transient and significant externalization of phosphatidylserine, which disappeared after longer incubation times. Apoptosis was then confirmed via the activation of executioner caspases 3/7 after spheroid exposure to PAM, as shown in Fig. 4D. Apoptosis was detectable after 6 h of exposure to PAM and increased over time. Interestingly, no apoptosis was observable with the osmolarity control (gas alone). It should be noted that the crown of detached and detaching cells is highly fragile. Thus, only *in situ* wide-field fluorescence microscopy within the cell culture dish was performed to assess propidium iodide penetration and apoptosis induction. This means that fluorescence coming from the whole spheroid (cohesive core + crown of detached cells) was detected. To assess the modification of the cohesive core induced by PAM 24 h post-treatment, spheroids were gently pipetted to allow removal of the crown of detached cells (Fig. 4E). Under both the control and PAM conditions, the remaining core of the spheroids did not present a loss of plasma membrane integrity or apoptosis induction, highlighting its viability. These results provide evidence that cells constituting the crown of detached cells underwent PAM-induced apoptosis and necrosis. This result corresponds to the decrease in size observed 24 h after exposure to PAM on the growth curve (Fig. 1B).

The concomitant occurrence of apoptosis and loss of membrane integrity indicates that PAM treatment induced both apoptosis and necrosis in spheroids. Apoptosis and necrosis were shown to be limited to cells detaching from the spheroid surface, namely, those in close contact with PAM. To amplify the destruction of tumor spheroids and target deeper layers, we performed successive treatments with PAM.

### Successive treatments induced a cumulative effect on the growth delay of spheroids

We showed that within 24 h, spheroids exposed to PAM presented a crown of necrosis and apoptosis-dying cells and a viable cohesive core. We hypothesize that successive exposure to fresh PAM would help stop tumor growth and/or destroy tumor spheroids.

**Figure 5:**
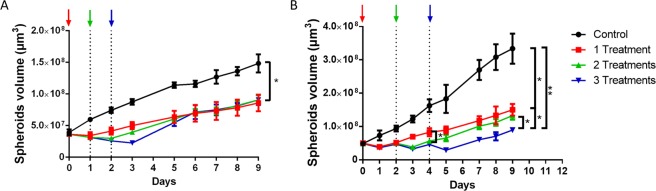
Successive treatments with PAM display cumulative effects on reducing tumor spheroid growth. At least 6 independent spheroids underwent a unique treatment with PAM (red squares) or two treatments (green triangles) or three successive treatments (blue inverted triangles), compared to control spheroid growth (black circles). Colored arrows indicate day of treatment with freshly produced PAM. (**A**) Treatments were performed every day. (**B**) Treatments were performed two days apart. *p < 0.05, **p < 0.01.

For that purpose, batches of spheroids were exposed to fresh PAM one, two or three times, and their growth was followed over 9 days. Figure 5 shows the volumes of spheroids after they underwent treatment every day (Fig. 5A) or every two days (Fig. 5B). Successive treatments performed every day (Fig. 5A) showed that although a volume loss is present the day following treatment, after 5 days, each condition (from one to three successive treatments) yielded the same spheroid volume. This result suggested that successive treatment one day apart is not sufficient to increase the PAM anticancer effect more than one treatment does.

In a similar manner, when performing successive treatment two days apart (Fig. 5B), after each exposure to fresh PAM, a reduction in spheroid size was observed, but growth started again within the following 24 h. Two successive exposures helped maintain tumor volume, but growth recovered 24 h later. Encouragingly, three successive treatments reduced the spheroid volume to less than the original volume. Spheroids treated successively 3 times were significantly smaller than those treated only twice. This experiment demonstrates a cumulative effect of successive PAM treatments with the possibility of not only limiting tumor spheroid growth but also reducing tumor size. This *in vitro* result demonstrated the importance of waiting for cell death mechanisms to be induced and completed before performing subsequent PAM treatment.

## Discussion

**Figure 6:**
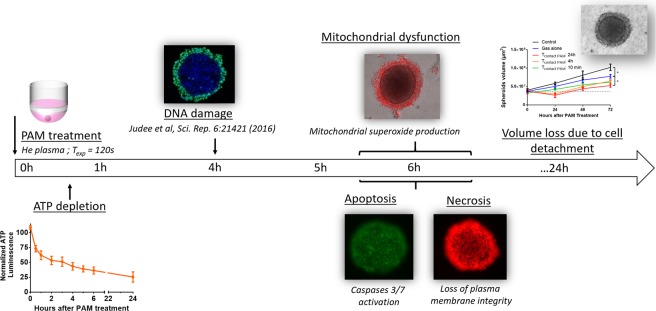
Cascade of events leading to cell death and tumor volume reduction after PAM treatment *in vitro*.

The aim of this work was to better elucidate the effects of PAM on human 3D colorectal tumor spheroids by describing the *in vitro*-induced cell death kinetics and mechanisms to improve its *in vivo* therapeutic potential. The main cellular steps are summarized in Fig. 6.

We have historically made the choice to activate cell culture medium with cold plasma, and we performed a detailed assessment of the medium by several complementary analytical techniques to determine the different reactive oxygen species, such as hydroxyl radical, superoxide anion, and singlet oxygen, and nitrogen species produced^14^, both in water and cell culture medium. We observed that the composition of the medium significantly impacted the pH of the solution during plasma treatment, as well as the stability and reactivity with biomolecules of the different ROS and RONS produced. However, it should be noted that several other types of liquids, such as water and PBS, have been plasma-activated, characterized in terms of the reactive species produced, and tested in terms of anticancer properties *in vitro* or *in vivo*^41–43^. These two liquids may be more suitable for translational applications in clinics. Most of the reactive oxygen and nitrogen species are found in all these plasma-activated liquids but in different quantities depending on the liquid and the plasma experimental set up used.

We previously showed that PAM displayed a genotoxic effect on human tumor cells, mainly by the production of reactive oxygen species such as hydrogen peroxide (H_2_O_2_) and the generation of reactive nitrogen species^4,13,14^. Hydrogen peroxide is well known for its ability to break DNA double strands^4,13^. However, studies with spheroids treated only with H_2_O_2_ solutions in culture medium demonstrated that H_2_O_2_ alone cannot induce a long-term effect, even if it generates DNA damage^4,20^. Although H_2_O_2_ is necessary for PAM anticancer properties, other species involved in PAM play a key role in cancer cell death. Interestingly, we previously reported that the addition of superoxide dismutase (SOD) to PAM reduced DNA damage in the outer layers of spheroids, highlighting the key role of superoxide^13^. These results indicate that several species found in PAM play complementary roles in cellular behaviors.

Others studies further investigated the mechanisms induced at the cellular scale. Oxidative stress was shown to induce intracellular zinc (Zn^2+^) release and provoke Zn^2+^-dependent cell death in human neuroblastoma SH-SY5Y cells^44^, while normal human fibroblasts were less susceptible to PAM cytotoxicity, probably because cells with low intracellular mobile Zn^2+^ are less susceptible to PAM cytotoxicity^45^. Indeed, numerous recently published studies have reported that PAM specifically affects tumor cells while sparing normal cells. We previously demonstrated that PAM induced selective DNA damage in HCT-116 3D spheroids compared with normal human fibroblast GM637 spheroids^4^. In another study, it was shown that an optimal dose of PAM is able to selectively induce significant hepatocarcinoma cell apoptosis while minimizing damage to co-cultured normal hepatic cell lines^46^. This study confirms results obtained previously in two human epithelial ovarian carcinoma cell lines and normal fibroblasts. When treated with PAM, both types of epithelial ovarian carcinoma cell lines were discriminately killed through enhanced apoptosis induction, while normal fibroblast cells were not damaged^47^. These encouraging results demonstrate that PAM could be a promising tool for anti-cancer therapy while sparing normal surrounding tissues.

The recognized luminescence assay^48^ was used to quantify intracellular ATP content after exposure to PAM. We proved that dramatic intracellular ATP depletion occurred early (within 1 h) after exposure to PAM. The finding that no ATP was measured in the supernatants indicated that ATP loss occurred due to an intracellular mechanism. Our observations are consistent with those of other groups for cells exposed to plasma-activated liquids^45,49^. Interestingly, to the best of our knowledge, a distinct effect was observed solely with cells directly exposed to cold plasmas, which led to ATP extracellular secretion^50,51^.

Since PAM triggers cellular damage solely in the outer layers of spheroids while sparing the core, we suggested the application of successive treatments to improve anti-tumor efficacy in *in vivo* antitumor treatment perspective. However, the duration between each new treatment proved to be important. Indeed, treatment every day did not show any benefits, while treatment every two days led to tumor regression. Since the induction of cell death is a multistep process, it seems important to allow time for cells to go through all of them before applying a new treatment. In cells grown in monolayer, PAM was previously shown to have an anti-tumor effect on epithelial ovarian carcinoma chemo-resistant cells by inducing caspases 3/7 activation within 4 h after treatment^27^. In monolayer, PAM does not need to diffuse throughout the spheroid’s volume, meaning that all the cells grown on a flat surface were homogenously and directly exposed to PAM. This notion could explain why the apoptosis kinetics were faster in monolayers and underline the importance of successive treatments when targeting tissues in 3D.

To date, all *in vivo* studies with PAM^27,52,53^ have demonstrated a promising effect as an antitumor treatment in different types (solid, metastatic, liquid) of tumors. Indeed, after successive *in vivo* injection of fresh PAM, the growth of solid tumors was reduced, showing the anticancer effect of PAM in a murine model^27^. In this study, the authors showed a reduced tumor growth speed that confirmed *in vivo* anti-tumor effects of PAM in nude mice bearing ovarian cancer cells embedded in Matrigel^27^. Interestingly, some *in vivo* studies evaluated the efficacy of PAM administered intraperitoneally (distinct from intratumoral injections) in inhibiting peritoneal metastasis and showed a 60% decrease in the formation of peritoneal metastatic nodules^54^. Another group examined the effect of PAM on lymphoplasmacytic lymphoma cell lines and found that PAM induced plasma cell differentiation and reduced the tumorigenic population^55^. A next step for *in vivo* treatment with PAM is to use mice with a functional immune system, as it has been shown that immunogenic cell death and the stimulation of macrophages can improve the PAM effect^56,57^.

In conclusion, plasma-activated liquids may represent a novel, safe and efficient therapeutic method for the treatment of peritoneal metastases^54^, blood cancers^55^ and solid tumors. Furthermore, PAM preferentially activates cell death modalities, including apoptosis and necrosis, in tumor cell lines and primary cancerous cells compared with their normal counterparts. Thus, this therapeutic modality seems to offer the selectivity that is lacking in many other available treatments.

## Materials and Methods

### Low-temperature helium plasma jet production at atmospheric pressure

The plasma jet device is based on a dielectric barrier discharge configuration as previously described^4,13,14,20^. Two aluminum electrodes are wrapped around a quartz tube, and a generator delivers high-voltage mono-polar square pulses between them. The characteristics of the power supply are as follow: 10-kV voltage, 10-kHz frequency and 1-µs pulse duration. Helium is delivered through the quartz tube with a flow rate of 3 L/min.

### Plasma-activated medium production

PAM was produced by exposure of 100 µL of Dulbecco’s Modified Eagle’s Medium (DMEM) without pyruvate (Thermo Fisher Scientific, #41965) in 96-well plates to a low-temperature plasma jet. Cell culture medium was exposed to the plasma jet for 120 seconds. All PAM production was performed under the same experimental setup (applied voltage, frequency, pulse duration and gas flow) and at the same distance between the plasma jet tube output and the upper surface of the liquid medium, i.e., 2 cm. PAM was freshly produced when needed for the experiments; it was not stored before use.

### Cell culture

Human colorectal carcinoma cell line HCT-116 was purchased from ATCC (# CCL-247). These cells were chosen for their ability to produce tumor spheroids^4,58,59^. Cells were grown in DMEM containing glucose, L-glutamine and pyruvate (Thermo Fisher Scientific, #41966), supplemented with 10% (v/v) heat-inactivated fetal calf serum, 100 U/ml penicillin and 100 µg/ml streptomycin. Cells were cultured at 37 °C in a humidified atmosphere containing 5% CO_2_. Cells were regularly tested (every two weeks) for mycoplasma contamination using the MycoAlert Mycoplasma detection kit (Lonza).

### Spheroid generation

For spheroid production, 500 cells were seeded in ultralow attachment 96-well plates with a round bottom (Corning, Fisher Scientific) and allowed to grow for 5 days in DMEM with glucose, L-glutamine and pyruvate (Thermo Fisher Scientific, #41966), supplemented with 10% (v/v) heat-inactivated fetal calf serum, 100 U/ml penicillin and 100 µg/ml streptomycin in the incubator before use as previously described^4,58^.

### Spheroid treatment with PAM

Three distinct contact times (T_contact_) of spheroids with PAM were applied: 10 min, 4 h and 24 h. Briefly, after PAM production, spheroids were immersed in 96-well plates in 80 µl of freshly produced PAM for 10 min, 4 h or 24 h. After exposure to PAM, they were then immersed in fresh non-activated complete cell culture medium for the remaining culture time.

In the case of successive treatments, spheroids were exposed to freshly produced PAM once, two times or three times at one- or two-day intervals and continuously cultivated within this PAM. Their growth curve was followed over 9 days as described below.

### Osmolarity and pH measurements

Osmolarity of PAM was measured using a cryoscopic osmometer (Osmomat 030; Gonotec). The osmometer was calibrated using 50 µL of solution of known osmolarity of 300 mosmol/kg. A total of 50 µL of three independent samples were then measured for each condition (DMEM, PAM, He only). The pH of these solutions was measured using a microprocessor pH meter (pH 210) from Hanna Instruments from three independent samples for each condition (DMEM, PAM, He only).

### Spheroid growth curve analysis

Growth of spheroids was followed by taking photographs with a wide-field light microscope before treatment and regularly after treatment for 3 or 9 days depending on the experiments using a Leica DMIRB microscope coupled to a coolSNAP HQ camera (Roper Scientific, Photometrics). The volume of the spheroids was determined following average diameter measurements using ImageJ software (NIH, Bethesda, USA) as previously described^4,13,20^.

### ATP quantification

Cell viability in spheroids was determined using the CellTiter-Glo® 3D Cell Viability Assay (Promega) according to the manufacturer’s instructions. Briefly, spheroids were incubated with 80 µl of PAM for 24 h in a 96-well white plate. At different time points, the same volume of reagent (i.e., 80 µl) was added to the well and incubated at room temperature under strong agitation for 30 min. Then, luminescence was read with a microplate reader (CLARIOstar, BMG LabTech). Alternatively, spheroids were manually removed from wells to quantify ATP released in the PAM supernatant. A similar procedure to that described above was applied, namely, addition of the same volume of reagent, incubation under agitation at room temperature and luminescence quantification with a microplate reader. Finally, to assess whether reactive species generated in PAM affect ATP integrity, an experiment was performed with a standard solution of ATP (10 nM) incubated over 6 h in PAM.

### Mitochondrial dysfunction detection

MitoSOX Red Mitochondrial SuperOxide indicator is a fluorogenic dye used for selective detection of superoxide in the mitochondria of living cells. Once in the mitochondria, this reagent is oxidized by superoxide to exhibit red fluorescence. Spheroids were immersed in 5 µM of MitoSOX reagent solution and incubated for 10 min at 37 °C, protected from light. MitoSOX fluorescence (λ_ex_ = 510; λ_em_ = 580 nm) was observed within spheroids using a Leica DMIRB microscope coupled to a coolSNAP HQ camera (Roper Scientific, Photometrics). ImageJ software was used to process the fluorescence images.

### Plasma membrane integrity determination

Propidium iodide is a probe that becomes highly fluorescent when intercalated within DNA^60^. However, it is a non-permeant probe, meaning it will only penetrate inside cells presenting defects in plasma membrane integrity. Briefly, at different time points after exposure to PAM, propidium iodide at a final concentration of 100 µM was added to the wells to label cells presenting a defect in plasma membrane integrity. Propidium iodide fluorescence (λ_ex_ = 538; λ_em_ = 617 nm) was assessed with a Leica DMIRB microscope coupled to a coolSNAP HQ camera (Roper Scientific, Photometrics). ImageJ software was used to process fluorescence images. To quantify live propidium iodide penetration over time, spheroids exposed to PAM and 1 µM propidium iodide were placed within an IncuCyte ZOOM live cell imaging system (Essen Bioscience). Acquisitions were obtained every 30 min for one day, and quantifications were performed with the software associated with the videomicroscope. A movie demonstrating propidium iodide penetration into spheroids exposed to PAM is available as Movie 1.

### Apoptosis detection

The Image-iT™ LIVE Red Caspase-3 and -7 Detection Kit from Molecular Probes was used according to the manufacturer’s protocol to visualize, by fluorescence microscopy, apoptosis induction in spheroids over time after exposure to PAM. Briefly, at different time points, spheroids exposed to PAM were incubated with FLICA kit reagent for 1 h in an incubator at 37 °C. After three washing steps in a large volume of PBS, the red fluorescence signal detected with a Leica DMIRB microscope coupled to a coolSNAP HQ camera is a direct measure of the amount of active caspase 3/7 in the treated spheroid. For convenience, images of apoptosis induction will be presented in green instead of red. To corroborate cell death induction through apoptosis, we also performed flow cytometry analysis of isolated cells from spheroids to detect precocious externalization of phosphatidylserine, detected with an annexin V-FITC probe while being vigilant that cells remained negative to propidium iodide staining. After incubation with PAM (Tcontact = 1, 4 and 6 h), spheroids were gently dissociated for 6 min in trypsin at 37 °C. For each replicate, four spheroids were dissociated and pooled to obtain enough cells for flow cytometry. Cells were processed according to the manufacturer’s instructions (BioVision, Mountain View, CA, USA). Differences between values were assessed by one-way ANOVA followed by Dunnett’s multiple comparisons post-test.

### Statistical analysis

For every set of *in vitro* experiments, at least six biological replicates were analyzed. The reported data correspond to the means ± SEM of at least two independent experiments or to representative images from these experiments. Differences between data were assessed by Student’s t-test. Overall statistical significance was set at p < 0.05.

## Supplementary information

Movie 1
